# Her, His, and their journey with endometriosis: a qualitative study

**DOI:** 10.3389/fgwh.2024.1480060

**Published:** 2024-12-03

**Authors:** Shiri Shinan-Altman, Aya Wertheimer, Bat-El Frankel, Yaira Hamama-Raz

**Affiliations:** ^1^ The Louis and Gabi Weisfeld School of Social Work, Bar-Ilan University, Ramat Gan, Israel; ^2^ Endo Israel Community Association, Givatayim, Israel; ^3^ School of Social Work, Ariel University, Givatayim, Israel

**Keywords:** endometriosis, coping, qualitative method, dyadic interview analysis, couples

## Abstract

**Background:**

Endometriosis, impacting roughly 10% of reproductive-age women and girls globally, presents diagnostic challenges that can cause significant delays between symptom onset and medical confirmation. The aim of the current study was to explore the experience of women with endometriosis as well as that of their partners, from pre-diagnosis to diagnosis to post-diagnosis.

**Methods:**

In-depth semi-structured interviews were conducted with 10 couples coping with endometriosis. Each partner was interviewed separately, and each interview was analyzed both individually and as part of a dyad, using the dyadic interview analysis method.

**Results:**

Three main themes emerged: (i) “Relationship in the shadow of uncertainty”: Coping with health symptoms prior to the formal endometriosis diagnosis; (ii) Coping together or alone when receiving the endometriosis diagnosis; and (iii) “The day after the diagnosis”: Moving between adversity and growth.

**Conclusions:**

The study's findings emphasize the importance of viewing the diagnosis from a dyadic perspective and comprehensively – that is, from pre-diagnosis to accepting the formal diagnosis to post-diagnosis. This journey can have a profound impact on both couple members, affecting their day-to-day functioning, communication, emotional and physical intimacy, and fertility.

## Introduction

*Endometriosis* is a disease in which tissue similar to the lining of the uterus grows outside the uterus (1). It affects roughly 10% (190 million) of reproductive-age women and girls globally and can cause infertility (2). According to the WHO (1), endometriosis symptoms include a combination of painful menstrual periods, chronic pelvic pain, pain during and/or after sexual intercourse, painful bowel movements, painful urination, fatigue, depression or anxiety, abdominal bloating, and nausea. Given the variability and broad symptoms involved, endometriosis is not easily diagnosed by healthcare workers, potentially causing a lengthy delay between symptom onset and diagnosis (3). Diagnosis can take seven years on average after symptom onset (4) but ranges from four to 11 years (5), suggesting a major impact on women's psychological well-being both before diagnosis and after (6). Indeed, a recent meta-ethnography in which the illness experience of endometriosis patients across the globe was investigated stressed the negative impact of this disease on many aspects of life (7). Specifically, the authors found that women with endometriosis encountered a multitude of problems including infertility, difficulty in maintaining intimate relationships (both emotional and physical) with their partners, psychological distress, negative emotions that could lead to suicidal ideation, low sense of self, low productivity at work, increased absenteeism, poor academic performance, social isolation, and low quality of life (7).

In Israel, the recognition and management of endometriosis have received growing attention at both policy and societal levels in recent years. The Israeli Ministry of Health has launched several initiatives aimed at raising awareness and improving the diagnosis of endometriosis through public health campaigns, with a focus on early detection and patient education (8). In this context, Wertheimer and colleagues (9) found that the average delay in diagnosing endometriosis in Israel is 11.2 years, which can be divided into two phases. The first phase involves an average delay of 4.6 years from the onset of symptoms to the point of reporting them to a medical professional, while the second phase includes an average delay of 6.6 years from the initial reporting to receiving a formal diagnosis. Their study also revealed that younger women experienced shorter diagnostic delays. Notably, only about 42% of the women reported that a medical professional was the first to raise the suspicion of endometriosis. The remaining women became aware of the possibility of endometriosis through media exposure, the activities of associations, independent online research, or conversations with acquaintances (9). Today, endometriosis is included in the basket of health services provided under national health insurance, offering more comprehensive and accessible treatment options for affected women (10).

In addition to endometriosis's impact on the woman's life, it also has an impact on the woman's partner's life. The negative emotions that have been reported among patients' partners include depression, anxiety, helplessness, frustration, worry, and anger (11–13). Additionally, partners of women with endometriosis have reported negative effects on their sex life (i.e., lack of communication about sexuality, sexual dysfunction, avoidance of sexual intercourse), intimacy, and the couple's relationship (14, 15).

Moreover, as endometriosis is a shared experience within the couple (16), several researchers have explored dyadic relations and marital satisfaction in the context of endometriosis (15–18). For example, Facchin and colleagues (17) revealed that women who perceived their partner as being more interested and actively involved in the management of their endometriosis (for instance by accompanying them to medical visits) reported greater relational satisfaction and better dyadic coping.

Considering the impact of endometriosis on both spouses, it is important to recognize that Israel's cultural landscape is heavily shaped by traditional values and societal expectations surrounding marriage, family, and procreation (19). There is a particularly strong emphasis on family life and childbearing, with significant social pressure on couples to have children soon after marriage (20). This cultural expectation often intensifies the emotional and psychological distress experienced by women diagnosed with endometriosis, who may already be struggling with issues related to infertility and intimacy (21). Partners may also feel an increased sense of responsibility to support their spouses, reflecting cultural notions of marital solidarity and traditional gender roles (13).

Taken together, it seems clear that endometriosis is a physically and mentally debilitating disease, with high emotional costs for both patients and partners (18). However, less is known about patients' and partners' pre-diagnosis experience, and whether the pre-diagnosis period impacts the acceptance of the diagnosis as well as the post–diagnosis period. Based on the Biopsychosocial Model (22), which emphasizes the complex interactions between biological, psychological, and social factors in understanding health and illness, we aimed to explore the experience of patients and their partners throughout their endometriosis “journey”—from pre-diagnosis, to receiving the diagnosis, to post-diagnosis management. By adopting this model, our study acknowledges that the experience of endometriosis extends beyond physical symptoms, impacting intimate relationships, self-identity, and social participation (23). Gaining a deeper understanding of the experiences of both couple members will provide professionals in the field with knowledge regarding the design of suitable interventions addressing both patients' and partners' needs.

## Method

In this study we used a qualitative-phenomenological approach (24), exploring participants' experiences with endometriosis so as to understand the phenomenon. This approach allows researchers to gain a rich understanding of the interviewees and uncover meaningful insights about the complex phenomenon under study (25). By focusing on participants’ lived experiences, phenomenological inquiry seeks to capture the essence of how individuals perceive and make sense of their condition, providing depth and context to their emotional and relational responses (25).

### Sample

Purposive sampling (26) was used, and the inclusion criteria were as follows: (1) having received a diagnosis of endometriosis at least nine months prior to the study, (2) being aged 18 and above, and (3) being in a heterosexual cohabiting relationship for at least one year. Given the dyadic nature of the study, both partners were asked for consent to participate.

The sample included 10 dyads (20 participants) aged 21–45 (men aged 21–39; women aged 22–45). Five couples were married, and two of them also had children. Years of education for all participants ranged between 12 and 20. Time elapsed since the endometriosis diagnosis ranged from 9 months to 17 years. Three couples were in a cohabiting relationship only post-diagnosis (for more details see Table 1).

**Table 1:** Demographic characteristics of participants (*n* = 20).

Dyad number	Names	Gender	Age	Years of education	Marital status	Number of children	Time since diagnosis	Other chronic disease
1	Gili	Female	32	18	Not married	0	9 months	Diabetes, psoriasis
	Tom	Male	28	15			–	–
2	Jenny	Female	45	16	Married	2	17 years	Fibromyalgia, allergy
	Jacob	Male	39	17				
3	Mili	Female	22	13	Not married	0	2 years	Insomnia
	Robert	Male	21	12			–	–
4	Lili	Female	31	15	Married	0	3 months	–
	Ron	Male	32	17			–	–
5	Su	Female	23	15	Not married	0	3 years	Polycystic ovaries, arthritis, adenomyosis
	Jack	Male	25	12			–	–
6	Amy	Female	23	12	Not married	0	1 year	Fibromyalgia
	Dan	Male	22	12			–	–
7	Dorin	Female	27	15	Married	0	1.5 years	–
	Moshe	Male	27	15			–	–
8	Mika	Female	28	12	Not married	0	3 years	Irritable bowel syndrome
	Ben	Male	28	12				
9	Rivi	Female	37	15	Married	7	3 years	–
	Yuval	Male	39	15			–	–
10	Maya	Female	34	18	Married	0	1 year	–
	Eran	Male	37	20				–

### Procedure

After obtaining approval from the Ethics Committee (Authorization No. 072202), participants were recruited from August to October 2022 through the Endo Israel Community Association, which advertised the study on its Facebook and Instagram pages. Because the data was collected via social networks, we could not assess the response rate.

The interviews were conducted by five female interviewers, four of whom had themselves been diagnosed with endometriosis and who served as members of the Endo Israel Community Association (the first author was the fifth interviewer). All interviewers had prior training in qualitative interviewing including the conducting of mock interviews prior to the start of this study. To ensure consistency across interviews, an ongoing supervision and mentoring regarding interviewing style were provided by S.S.A., a social worker with extensive experience in qualitative research. Debriefing sessions were held throughout the data collection period to ensure alignment in interview techniques and adherence to the research objectives.

The interviewers contacted each patient and partner by phone to explain the study's aims and procedure. Once both partners agreed to participate, the interviewers scheduled a separate interview with each one. The same interviewer interviewed both members of each couple. For ethical reasons, each participant received information regarding the purpose of the study and signed a letter of consent that included an explanation stating that data would be used for research purposes only and that confidentiality would be preserved by changing identifying details. Participants were informed they could withdraw from the study at any time and could refuse to answer any question. None of the participants dropped out. No incentives were offered. Each interview was conducted via Zoom and lasted 60–90 min. Each partner was interviewed separately, and each interview was analyzed individually and as part of a dyad. Notably, to address the ethical complexities of dyadic research, we followed recommendations from the literature (27) to ensure confidentiality. We anonymized sensitive information and informed participants that the analysis would prevent partners from identifying each other's disclosures.

The interviews were conducted in Hebrew, recorded, and then translated into English. Each translation was reviewed by two native speakers, including a professional translator. Data collection continued until theoretical saturation was achieved, meaning that additional interviews no longer produced new material for analysis (17).

### Research tools and instruments

The study's qualitative data were collected through in-depth semi-structured interviews (28). The research team opted for interviews as the primary method to explore and understand participants' experiences (see Appendices 1, 2). The interviews followed a flexible guide that included significant key areas to facilitate a dialogue between the interviewer and the interviewee and enable meaningful self-expression (25). We developed the interview guide based on a phenomenological framework, designed to explore participants' lived experiences of endometriosis and its impact on their relationships. The guide included open-ended questions aimed at facilitating an in-depth exploration of emotional, physical, and relational aspects of coping with the disease.

### Data analysis

The dyadic interview analysis method (29) was used to analyze the data. This method involves following the initial steps of thematic analysis, such as reading and re-reading the interviews, making notes, and coding the data to identify units of meaning and their corresponding titles. This process was repeated for each partner's interview, and all 20 interviews were analyzed. The analysis focused on the endometriosis diagnosis journey, resulting in three themes. The analysis, conducted by S.S.A., was conducted on both individual and dyadic levels to examine the data from different perspectives (30).

*At the individual level*, each partner's quotes were analyzed in an in-depth fashion, with content, structure, linguistics, grammar, and metaphors being taken into consideration (31). This process involves deriving interpretations directly from the data. The same analysis was conducted for quotes from the other partner. *At the dyadic level*, the focus was on examining the contrasts and overlaps between the partners' quotes. Doing so involved exploring the text and subtext, as well as the partners’ descriptions and interpretations within each theme and sub-theme (29). The themes were identified on the basis of the content and structure of the quotes specifically related to the endometriosis diagnosis.

To ensure the trustworthiness of the study findings, the researchers employed multiple methods. These included conducting in-depth interviews for a comprehensive understanding of participants' experiences, verbatim transcription of interview data, and adhering to credibility criteria (i.e., presenting data in a dense and detailed manner). Triangulation through the use of two different sources of interviews and dyadic analysis further enhanced the trustworthiness of the data (32).

## Results

This section presents three key themes from interviews with women diagnosed with endometriosis and their partners. These themes explore the emotional, physical, and relational challenges experienced across the pre-diagnosis, diagnosis, and post-diagnosis stages, highlighting the impact of endometriosis on both individuals and their relationships.

### Theme 1: “Relationship in the shadow of uncertainty”: coping with health symptoms prior to the formal endometriosis diagnosis

The couples talked about a joint coping journey that started pre-diagnosis. This period was marked by an arduous quest for answers, as the disease's symptoms were taking a severe toll on the women's physical and emotional well-being. All of the women talked about the physical discomfort they had experienced in various regions of their bodies, to the extent that it hindered their daily functioning. Furthermore, the women, along with their partners, expressed a great deal of unease over the ambiguity and meaning of these symptoms. During this difficult period, the women in four couples began to distance themselves from their partners. The partners responded with understanding, recognizing the women's need for space and time to process their emotions. Despite the temporary distance between them, the partners remained committed to supporting each other (Boxes 1, 2).

BOX 1 **Navigating ambiguity and distance in the pre-diagnosis phase of endometriosis.**

**Table:** 

Rivi	Yuval
“*After giving birth, my body changed drastically. My periods became excruciatingly painful, and I experienced constant pain throughout my body. It was debilitating, and I couldn't even get out of bed in the morning. I felt confused and lost, not knowing what was happening to me. I spent a lot of time searching online for answers and trying to make sense of it all. During this period, I became so absorbed in my own struggles that I became disconnected from Yuval and the world around me.*”	“*It took us a long time, about a year, to realize it was endometriosis. We, including the doctors, were confused. Seeing her in pain was tough, so I gave her space, as she needed it from me and the children.*”

BOX 2 **Balancing solitude and connection in the pre-diagnosis phase.**

**Table:** 

Mika	Ben
“*During this period I preferred to be alone… I didn't share with Ben what I was going through. … I went to the doctors alone, cried alone… I felt that it was mine and that no one could understand me. I think in retrospect that it must have been difficult for him (Ben) but honestly I was concentrating on myself.*”	“*She was very aggressive in the period before the diagnosis, really attacking me verbally and I get defensive in such situations… It's not that we didn't love each other during that time but we were distant from each other… mainly because of her… She preferred to be alone and I respected that even though I wanted to be there for her more… I think it's also because she thought I couldn't understand her, after all the symptoms she was experiencing were female-related*”

The individual coping within the dyad during the pre-diagnosis period was reflected in five of the women preferring to handle their emotions and cope with the situation alone, rather than burdening their partners: They often chose to undergo medical tests on their own and refrained from sharing their emotional struggles with their significant others. Five male partners discussed the dilemma regarding the emotional distance that was created between them as a result: They expressed their wish to support their female partners and be part of the process but also acknowledged their partners’ need for space. Despite the emotional difficulty and their limited understanding of the situation, given the female nature of the as-yet undiagnosed disease, five partners emphasized that they would have preferred to be involved in the process.

The “we” of the experience even in the pre-diagnosis phase was expressed among five dyads who described that due to how significantly the disease was impacting the women's functioning, the couple together tried to find a name for it (i.e., a diagnosis), on the basis of the women's physical and emotional symptoms. One of the most prominent features during this period was running from doctor to doctor together, a feeling of uncertainty, and a lack of control in the face of various proposed diagnoses, some of which in retrospect were wrong and only led to more frustration. The couples described a common experience around the search for a diagnosis; both the women and their partners described a shared marital experience (Box 3).

BOX 3 **Shared journey: seeking answers in pre-diagnosis.**

**Table:** 

Gili	Tom
“*We went to see gastroenterologists and a few gynecologists to figure out what was wrong. They didn't think it was endometriosis, but then we learned about doctors on Facebook who specialized in this area and could diagnose it. We felt lost and helpless during this time, like we were losing control. Finally, when we discovered that it was endometriosis, it brought us a sense of relief as a couple. It gave us an anchor in the storm and a name for what we were facing.*”	“*It was a turbulent time for us as a couple as well, we really suffered… and I can say it was hard for me to see her like this… a year of pain until we received the diagnosis… a disease with an actual name, endometriosis. She, me, and us as a couple had some relief… but up until the diagnosis, our relationship was living in the shadow of uncertainty.*”

Lili and Ron (Box 4) also described a joint couple experience on the way to receiving a diagnosis. Their shared experience could be described as “we're in it together for better and worse.” Their relationship experience during this period of uncertainty was enveloped in mutual loyalty and commitment.

BOX 4 **Loyalty and commitment in diagnostic uncertainty.**

**Table:** 

Lili	Ron
“*Ron has been my rock throughout this journey. He's been there for every doctor's visit and examination, supporting me every step of the way. When I received the diagnosis, he showed a vulnerable side, shedding tears and worrying about my health. It strengthened our bond, and I realized that he's there for me no matter what.*”	“*She felt pain, severe pain in the pelvic area… We ran straight to the hospital for all kinds of tests, until one of the doctors diagnosed it as endometriosis… we were together the whole time… loyal and committed to each other… After we realized that it was a disease, Lili was able to explain to me more about what she was going through.*”

Before receiving the diagnosis, six women experienced internal conflict. They wanted to understand whether there was a name for their physical symptoms and whether it was recognized in the medical literature. They felt frustrated and confused that their symptoms did not seem to be taken seriously by healthcare providers or society. However, they also feared the potential consequences of receiving a diagnosis and worried about how it might impact their lives. This inner turmoil caused them to feel guilty for not facing the situation and not allowing their partners to be more involved. The partners, on the other hand, were simply “hungry” for information; they wished to fully comprehend their partner's health status, educating themselves about the disease they were facing (Box 5).

BOX 5 **Inner conflict and the quest for answers.**

**Table:** 

Maya	Eran
“*The uncertainty of whether to seek a diagnosis or not felt like walking on a tightrope. There was fear that naming the condition would have consequences, and maybe it would be a serious illness. But then I thought about my partner and it didn't seem fair that he had to experience the symptoms while I avoided knowing what they meant. This struggle between getting a diagnosis or not caused me confusion, anger, and self-blame.*”	“*I'm a rational type… looking for a logical explanation for everything… reading, studying… that's why I wanted to know what she had… what it was called, what the diagnosis was… not only for myself of course but also for her… with a diagnosis you can know what it's about and start acting accordingly… I know she had a hard time making a decision about this [receiving a diagnosis].*”

### Theme 2: Coping together or alone when receiving the endometriosis diagnosis

This theme pertains to the experience of receiving an endometriosis diagnosis after a prolonged period of waiting. Six women were with their partners when they received the diagnosis, and four women were not in a relationship at the time of the diagnosis. Therefore, the latter group of women had to break the news about the disease to their partners (i.e., when they entered these relationships). The couples who were together described a common experience of relief upon receiving the diagnosis and the mutual support made possible by the partner's presence (i.e., he too could hear what the doctor had to say about the disease). Even though endometriosis is a “female disease,” it also directly affects the partner in various ways, thus making the partner's presence meaningful (Box 6).

BOX 6 **A common experience of relief upon receiving the diagnosis.**

**Table:** 

Lili	Ron
“*After the doctor gave it a name, we both sat down and began to understand… we asked questions, we were interested, and the doctor allowed us to ask everything… it was so meaningful for us, a certain relief… we were even able to see a light at the end of the tunnel because we understood from the doctor how to deal with various challenges… for example, sex… we understood that the disease is for life, a chronic disease, but there is a lot that we can do about it…*”	“*We also started asking a lot of questions while he was explaining things to us. I'm a very technical person, so when I don't know something, I deeply analyze it. So, I asked the doctor, “On a scale from one to ten, how bad is it? What are the dangers, on a scale from one and ten?” without too much emotion. I asked him to explain how we should deal with various challenges… I was a participant in the conversation and it was meaningful for me.*”

For four couples, receiving the diagnosis was accompanied by feelings of stress and fear. They struggled between hiding their feelings, in order to appear strong in the face of this new information, and revealing their true feelings/fear (Box 7).

BOX 7 **Emotional responses to receiving the diagnosis.**

**Table:** 

Dorin	Moshe
“*When we left the doctor after the diagnosis Moshe told me that it didn't matter what he felt; only what I felt… and we went down the stairs and I really cried… I was afraid of what I would have to face now that we knew what it was and maybe it wasn't good that I was crying like that… maybe it was better to look strong, for me but also for him.*”	“*When we were sitting in the doctor's office I felt pain, maybe because we had waited so long to understand what she had… it was an emotional pain, maybe it was a reaction to Dorin's physical pain, her suffering and what she went through to finally understand what she had… the doctor was very attentive. He was also sensitive to me and to my questions.*”

Other couples talked about being together but feeling apart when they received the news. Physically they were both present in the doctor's office, but emotionally/psychologically each was present in the situation mainly with himself/herself (Box 8).

BOX 8 **Being together but feeling apart when receiving the diagnosis.**

**Table:** 

Mika	Ben
“*I felt terribly alone… I realized that only I could really help myself, and that I couldn't expect anything from anyone, and that I would have to take responsibility for myself… I mean, I was happy that he [Ben] was sitting next to me and listening to the doctor, but in my feelings, I was alone… me and the disease.*”	“*The doctor spoke, and I felt pressure, I didn't know what would happen, how she would manage with it, how I would manage with it, how we would manage with it together… I was thinking about her, about me, about us… however, the truth is that I didn't feel comfortable revealing my feelings, I'm not used to talking about feelings, I usually hide them.*”

Among three dyads, the women received the diagnosis before being in a cohabiting relationship, and therefore they were the ones who actually informed their partners about their disease. The delivery of this news was more complex for these women than the others, as they were concerned that the partner would fear or be alarmed by the disease and its implications for their relationship. Among these unmarried couples there was a gap between the woman's fear of telling her partner about the disease and the partner's desire to support and help his partner (Box 9).

BOX 9 **Navigating disclosure of endometriosis in emerging relationships.**

**Table:** 

Mili	Robert
“*I was afraid to tell him that I had endometriosis. To be in the situation of having to “spread the word”. For me it meant actually being in this situation for the second time, only now I was the messenger. And if he panicked? And if he ran away? I had many fears but somewhere or other I had enough confidence in our relationship that he would understand.*”	“*I was somewhat prepared for it because I knew she had female-related health issues. When she told me her diagnosis, I hugged her. I won't deny that I had some worries and occasional fears, but I'm here for her. I reassured her that I would be by her side, and I wouldn't ever leave her because of the disease.*”

### Theme 3: “*The day after the diagnosis”*: moving between adversity and growth

After the endometriosis diagnosis, both partners experienced relief at finally understanding the cause of the women's suffer. The women were pleased to learn they could receive treatment that would help reduce their symptoms, improving their quality of life and their ability to engage in sexual activity. Five partners also talked about feeling relieved that their female partner's condition was finally being addressed, and they felt more confident about providing support and care for her (Box 10).

BOX 10 **Understanding endometriosis and its impact.**

**Table:** 

Maya	Eran
“*The day after the diagnosis was a relief for both of us. It helped us understand what was happening to me and how it was impacting our relationship. It felt like finding a path forward because we discovered there were treatments that could help us. I'm currently trying new treatments to manage the pain and improve our sex life.*”	“*Today, now that we understand the disease Maya has, I feel more confident in helping her. For example, in sexual relations, I have a better understanding of the meaning of the disease and how it affects her sexual functioning. Our communication and openness really allow us to face the challenges.*”

Regarding the post-diagnosis period, six women also talked about having a new understanding of the challenges they faced in maintaining intimacy with their partners. They felt that the pain they experienced during intercourse – which made sex unpleasant or even impossible – now had more “legitimacy” (i.e., it now made more sense). Additionally, according to eight women, the physical and emotional toll of living with endometriosis led to feelings of anxiety, depression, and low self-esteem, which affected their ability to feel sexually confident and comfortable. It should be noted that the topic of sexual intimacy was discussed mainly by the women, and they went into detail and specifics; the male partners, however, discussed this issue in a limited and more general way. Five partners talked about their difficulty in understanding the extent of their partners’ pain and discomfort, although they knew that such pain/discomfort was part of the disease. They felt helpless or frustrated when their partner was in pain, especially if there was little they could do to help (Box 11).

BOX 11 **Navigating sexual and emotional challenges after an endometriosis diagnosis.**

**Table:** 

Amy	Dan
“*I understand that the pain during sex is attributed to the disease. Even though the consequences of the disease were explained to me at the diagnosis… I still feel difficulty both physically and mentally. A real lack of confidence in sexual relations, even though this is part of the prognosis.*”	“*The day after the diagnosis, I understand that part of the disease is living with pain and difficulty in having sex. The most frustrating thing is that I can't do much… I am considerate, of course, but the whole issue of sexual relations over time becomes complex.*”

Eight women described how in coping with the disease, they saw the opportunity for change and progress in their relationships, as a result of learning how to manage their physical and emotional symptoms. They sought and found effective pain management strategies such as medication, acupuncture, or physical therapy, and developed coping mechanisms to deal with the emotional toll of the condition. In addition, they learned to advocate for themselves in medical settings by asking questions, seeking second opinions, and exploring different treatment options. Their partners also saw the potential for change and progress. Their personal growth involved learning about the disease, its symptoms, and its impact on their partner's life, as well as providing emotional support and practical help. Partners’ personal growth was also reflected in their ability to learn to communicate effectively with their partner about their needs, feelings, and concerns, and finding ways to be a supportive and loving partner in the face of endometriosis (Box 12).

BOX 12 **Relationship transformation in coping with endometriosis.**

**Table:** 

Mili	Robert
“*I feel that today I am in a different, better place. As a couple too we know how to manage the symptoms of my disease together and I take part in treatments that will allow me and us as a couple to function.*”	“*I feel that I went through a process… I learned, I read about Mili's illness and I know how to communicate about this illness of hers, and this is how I feel I can support and help properly. It's a good feeling for me.*”

One factor the couples became aware of at diagnosis was that the disease could cause fertility problems. For seven of the women, fertility and bringing children into the world were seen as a fundamental part of their identity and sense of life fulfillment. Some of the women shared that the desire to have children was also tied to cultural and societal expectations, such as the expectation that they would become mothers at some point in their lives (Box 13).

BOX 13 **Fertility challenges in the face of endometriosis.**

**Table:** 

Gili	Tom
“*Losing the chance to have kids feels like a shattered dream. It's a personal goal I now know won't happen. My body can't handle pregnancy, and adoption isn't an option. It's disappointing because society expects me to have children, but luckily, my partner Tom is supportive of me. Still, it's undeniably a sad situation for me.*”	“*For some women, being a mother is a crucial part of who they are. But with endometriosis, it becomes a big obstacle. It's not like a broken bone or kidney problem; it directly impacts the ability to have children. Personally, it doesn't bother me much since I don't see myself as a parent, but it's still sad to know it's not even an option.*”

Table 2 presents the classification of main categories and subcategories.

**Table 2:** Classification of main categories and subcategories.

Main categories	Subcategories
*Theme 1: “Relationship in the shadow of uncertainty”*: Coping with health symptoms prior to the formal endometriosis diagnosis	*Joint journey of coping with pre-diagnosis symptoms: Couples navigated together the challenges of coping with endometriosis symptoms prior to diagnosis, with women experiencing physical discomfort and emotional distress, and partners providing understanding and support. *Struggles with the ambiguity of the symptoms and emotional turmoil*: The absence of a formal diagnosis created emotional turmoil as women dealt with frustration and self-blame, choosing to handle their emotions alone, while partners respected their need for space but desired more involvement. *Shared experience in the search for answers*: Couples faced uncertainty and frustration, running from doctor to doctor, but found solace in being together and sharing the journey, with partners expressing commitment, loyalty, and a desire to understand and support their female partners.
Theme 2: Coping together or alone when receiving the endometriosis diagnosis	*Mutual support and relief: Couples who were together during the diagnosis experienced a sense of relief upon receiving the diagnosis, as they could support each other and ask questions together, gaining a better understanding of the disease and how to cope with its challenges. *Internal conflict: *hiding vs. revealing feelings*: Some couples struggled with the dilemma of revealing their true feelings of stress and fear vs. appearing strong in front of their partner after receiving the diagnosis, leading to internal pressure and a conflict when responding to the news. *Subjective experience and individual coping: Despite physically being together, some couples felt emotionally distant from one another when receiving the diagnosis, with each partner mainly focused on their own thoughts and feelings, leading to a sense of being alone in their experience with the disease.
Theme 3: “The day after the diagnosis”: Moving between adversity and growth.	*Enhanced understanding and relief: The endometriosis diagnosis brought relief to both couple members and a shared understanding of the difficulties in their relationship that had stemmed from the disease, with the women feeling pleased that treatment options existed and the partners feeling more confident in providing support. *Intimacy challenges and emotional struggles: The women felt that the endometriosis diagnosis had given legitimacy to the pain they experienced during intercourse, while the partners recognized their limited ability to understand and expressed frustration when unable to alleviate their partner's pain. *Opportunities for growth and coping: The endometriosis diagnosis and coping with it offered an opportunity for relationship progress and personal development/growth. The women developed strategies to manage symptoms, and the partners learned to be more supportive, fostering progress in the relationship and overall well-being.

## Discussion

In this study we explored the individual coping within the dyad and dyadic experience of women with endometriosis as well as that of their partners in their journey of coping with the disease, from pre-diagnosis to receiving the diagnosis to post-diagnosis. The findings revealed that endometriosis has a profound impact not only on the women diagnosed but also on their partners and the couple as a whole. The diagnosis process and subsequent coping strategies are intertwined with emotional, relational, and societal factors, shaping how both partners experience and respond to the condition. The findings underscore the multifaceted nature of coping with endometriosis, which aligns with Engel's Biopsychosocial Model (22). This model posits that health and illness are influenced not just by biological factors but also by psychological and social dimensions. In this context, the coping mechanisms observed in both women with endometriosis and their partners illustrate the interconnected nature of emotional, relational, and societal factors in shaping responses to this chronic condition. This study highlights the need to view endometriosis as a shared experience within relationships, where both individual and dyadic coping mechanisms play crucial roles in navigating the challenges posed by the disease.

In the first theme, ‘*Relationship in the shadow of uncertainty*’*: Coping with health symptoms prior to the formal endometriosis diagnosis*”, the women presented a range of coping mechanisms, from detachment and handling their health symptoms on their own to recognizing the significance of collaboratively (i.e., with their partners), addressing the symptoms and their impacts. This duality between individualism and interdependence brought with it a spectrum of emotions, ranging from guilt and shame to feelings of acceptance and understanding that coping with these uniquely female symptoms needed to be a shared experience. In addition, the first theme's findings point to the long and painful road that the women and their partners had to travel in order to receive a formal diagnosis. Indeed, previous studies have shown the complexity of receiving a diagnosis of endometriosis (33, 34). The findings of the current study reinforce these earlier findings by showing that there are also emotional and marital implications resulting from the mere seeking of an accurate diagnosis. Pre-diagnosis, the current study participants experienced feelings of uncertainty and stress. According to several previous studies, stress in couple relationships is no longer conceptualized as an individual phenomenon but rather as a *dyadic stressor* (35, 36). Dyadic coping entails mutual involvement of both partners: providing and receiving support from each other and engaging in joint problem-solving activities and shared emotion regulation (37). In line with this notion, Bodenmann (35) coined the term “we-ness”, referring to the function of dyadic coping as enhancing mutual trust and intimacy, mutual attachment and commitment. In this context, the Biopsychosocial Model (22) suggests that emotional responses to health symptoms are not solely individual experiences but are also shaped by interpersonal and social dynamics. This highlights the importance of viewing the pre-diagnosis coping efforts within the broader context of the couple's relationship and the societal expectations surrounding gendered experiences of health. Yet in our study, some of the women preferred, during the pre-diagnosis phase, to cope with their symptoms individually, within the context of the relationship, rather than engaging in joint coping as a couple.

The second theme, “*Coping together or alone when receiving the endometriosis diagnosis*”, demonstrated how meaningful the presence of a partner during the diagnosis process was, as this presence enabled both partners to offer each other mutual support and also allowed the male partner to understand the meaning and consequences of the disease. Indeed, endometriosis is not solely a female disease; it also affects the partner in various ways, such as in terms of intimacy, fertility, and overall quality of life (38, 39). The presence of a partner during the diagnosis process can help alleviate some of the stress and anxiety associated with the disease and can facilitate open and honest communication about the disease's impact on the relationship.

The significance of mutual support during the diagnosis aligns with the Biopsychosocial Model's emphasis on the role of social and relational factors in health outcomes (22). The model helps elucidate how the presence of a partner not only provides emotional and instrumental support but also contributes to understanding the psychological burden of the diagnosis and the challenges in maintaining intimacy and communication in the face of a chronic illness like endometriosis.

Our findings also indicated that some couples struggled with a kind of tension between hiding their feelings upon receiving the diagnosis (so as to appear strong in the face of it) and revealing their true emotions/fears to their partner. This internal pressure and conflict between hiding vs. revealing feelings/fears may be related to societal expectations of how individuals should respond to a medical diagnosis, particularly as it pertains to gender roles and expectations. Studies have shown that gendered expectations can influence how individuals cope with illness and how they express their emotions [e.g., (40)]. Specifically, women are often expected to express their emotions more freely than men, who may be socialized to suppress their emotions and appear strong and stoic in the face of adversity.

In this context, the concept of “strength” as it applies to men can be further explored. Societal expectations often frame men as the 'strong figure' in relationships, responsible for providing support and maintaining composure. This notion was reflected in the coping strategies seen in our study, where men often emphasized rationality and technical approaches to understanding the diagnosis and managing their partner's health. These coping mechanisms align with existing research, which shows that men tend to use problem-solving and emotionally distanced approaches as a way of maintaining control in the face of stress (41). Such approaches, while seen as manifestations of strength, may also limit emotional expression, contributing to feelings of isolation within the couple dynamic (42).

Indeed, such notions may apply to the findings of the current study, in which couples felt pressure to hide their emotions and appear strong in front of their partners, and this was particularly the case for the men. However, hiding emotions and appearing strong may not always be helpful in coping with the emotional impact of a diagnosis, as it may lead to feelings of isolation and lack of support (43).

The third theme, ‘*The day after the diagnosis*’*: Moving between adversity and growth*”, highlights the dialectical lens, viewing the adversity that accompanies the endometriosis diagnosis and the personal/couple growth as potentially coexisting phenomena. Specifically, both members of the couple reported that initially they felt relief that the disease finally had a name, particularly as it took so long to receive a diagnosis. The women hoped that now their medical treatment would be accurately targeted, and that their partners would be better able to support/understand them. However, when the exact meaning of the disease became clear to both couple members, and they became more informed, their distress became apparent once again: Women had to deal with anxiety, depression, and low self-esteem pertaining to the various implications of their diagnosis, and their partners had to deal with hopelessness and difficulty in relieving their partners' pain. In this context, Missmer and colleagues (44) found that the most frequent symptoms that negatively affected various areas of the lives of women diagnosed with endometriosis, including their intimate relationships, were pelvic pain apart from menstruation, painful menstruation, and painful sexual intercourse.

Distress and concern about fertility, in light of the endometriosis diagnosis, also emerged among both couple members. Indeed, women with a history of endometriosis have twice the risk of infertility compared with women without a history of endometriosis (45). In this regard, Moradi and colleagues (46) indicated that the worrying about the possibility of infertility adds to the burden of endometriosis, and negatively affects psychological health, marital relationships, and social interactions, and causes feelings of stigmatization and hopelessness. Thus, it was not surprising that once the symptoms received the “endometriosis” label, dealing with fertility became a cause for additional distress. Nevertheless, some of the positive impacts of receiving the diagnosis – namely, personal and couple growth – also became clear. Women showed self-efficacy in terms of finding tailored and personalized treatments, in addition to standard care, and handling the medical setting in a more effective way. Their partners indicated that finally having a diagnosis strengthened their ability to communicate with their partner, as well as the intimate relationship itself. Our findings echo previous findings (13) regarding men's descriptions of how they live with their partners' endometriosis. The researchers of that study stressed several positive impacts including the development of a supportive approach to the partner which strengthened the relationship, becoming a more sympathetic person and a better partner, and being closer to the partner. These results suggest the possibility of posttraumatic growth (PTG), which is defined as the positive psychological change experienced as a result of a struggle with highly challenging life circumstances (47). In this regard, Marki and colleagues (48) revealed, via the use of focus groups with women who had a confirmed endometriosis diagnosis, that despite many difficulties and problems, women described positive impacts on their life, such as peace, patience, openness, personality development, and gratitude, after they had accepted the diagnosis.

The coexistence of adversity and growth within couples coping with endometriosis reflects the Biopsychosocial Model's framework (22), which recognizes that psychological resilience and adaptation are influenced by an interplay of biological challenges (e.g., physical symptoms), psychological responses (e.g., depression, anxiety), and social support structures (e.g., relational dynamics). This integrated perspective can help explain the potential for posttraumatic growth despite the substantial physical and emotional burdens posed by endometriosis.

The current study had a few limitations. First, the sample was composed mainly of heterosexual couples, with limited variability in socioeconomic status. Additionally, the study did not collect relevant demographic characteristics such as nationality, religion, levels of religiosity, or geographic distribution, which may have impacted the participants' experiences and the generalizability of the findings. Second, the endometriosis-specific information (e.g., disease stage/grading, confirmed diagnosis) was self-reported and not clinically confirmed. Third, most of the study's interviewers were members of the Endo Israel Community Association. Thus, their personal experience of both having the disease and conducting the interviews must be taken into consideration. We would recommend, going forward, that additional experiences among both endometriosis patients and their partners in a more heterogeneous sample be examined.

### Clinical implications

The research findings point to the importance of revising comprehensive treatment approaches for endometriosis; specifically, guidelines must be implemented for community physicians to expedite diagnosis, increase awareness, and normalize experiences. Doing so would help reduce diagnostic delays and provide support for individuals and couples. Individual and couple treatment protocols within endometriosis treatment centers, both in hospitals and in the community, should be developed, so as to address emotional needs and facilitate open communication. Additionally, healthcare professionals must deliver the diagnosis with sensitivity and provide information about the potential impacts of the disease on fertility, pregnancy, and childbirth, taking into consideration the couples' concerns. Offering information through workshops, virtual communities, lectures, and treatment centers can further enhance patient support.

### Conclusions

In this study the experiences of women with endometriosis and their partners, from pre-diagnosis to post-diagnosis, were explored. The findings revealed that coping with health symptoms before receiving a formal diagnosis involved balancing individualism and interdependence. Couples experienced uncertainty and stress during the pre-diagnosis period. The presence of a partner when receiving the diagnosis played a crucial role in providing support and facilitating communication about the impact of endometriosis on the relationship. However, couples faced challenges in expressing their emotions authentically during the diagnosis process. After receiving the diagnosis, couples felt relief that the disease finally had a name, but they also experienced distress and concern about pain and fertility issues. The study emphasizes the coexistence of adversity and growth, as well as the importance of mutual coping in addressing the psychological effects of endometriosis.

## Data Availability

The raw data supporting the conclusions of this article will be made available by the authors, without undue reservation.

## Appendix 1 Interview guide for women diagnosed with endometriosis

• Please go back to the time when your endometriosis was discovered. Tell me how it was discovered.
• How were you informed that you had endometriosis? Who was with you when you found out?
• Describe your relationship with your partner during the process of getting the diagnosis and in the period after it.
• Tell me about your partner's reactions to the endometriosis diagnosis. How did you feel about his reactions?
• If you were asked to describe your life before and after the discovery of endometriosis, what has changed? What has remained the same?
• What have you discovered about yourself and your partner as a result of the endometriosis diagnosis?
• If you were to divide into sections the way you coped with endometriosis from the beginning until now, how would you divide it? What name would you give each section?
• How has endometriosis affected/changed your relationship with your partner?
• In the eyes of your partner, how has endometriosis affected your relationship?
• In your opinion, how does endometriosis influence your intimate relationship with your partner?
• What helps you cope with endometriosis? How do you feel about this help?
• Tell me about your communication with your partner in regard to the consequences of endometriosis.
• When you bring up your fears and difficulties about your endometriosis in discussions with your partner, what are your thoughts and feelings?
• What advice would you give to other women diagnosed with endometriosis?
• What advice would you give to the partners of women diagnosed with endometriosis?

## Appendix 2 Interview guide for partners of women diagnosed with endometriosis

• At what stage of the relationship did you find out that your partner had endometriosis?
• How were you informed that your partner had endometriosis?
• When did the current relationship with your partner begin in relation to the disease and its symptoms?
• Describe to me the feelings and thoughts that went through your head immediately after receiving your partner's diagnosis.
• Describe your relationship with your partner during the process of receiving the diagnosis and in the period after it.
• How has endometriosis affected your relationship with your partner?
• In the eyes of your partner, how has endometriosis affected/changed your relationship?
• In your opinion, how does endometriosis influence your intimate relationship with your partner (both physical and emotional)?
• What helps you cope with endometriosis? How do you feel about this help?
• Tell me about your communication with your partner in regard to the consequences of endometriosis.
• When you bring up your fears and difficulties about endometriosis in discussions with your partner, what are your thoughts and feelings?
• What advice would you give to other partners of women diagnosed with endometriosis?
